# User experiences of structured stakeholder engagement to consider transferability: The TRANSFER approach

**DOI:** 10.1002/cl2.1284

**Published:** 2022-10-17

**Authors:** Heather Munthe‐Kaas, Heid Nøkleby, Sarah Rosenbaum

**Affiliations:** ^1^ Reviews and Health Technology Assessments Norwegian Institute of Public Health Oslo Norway; ^2^ Present address: Centre for Epidemic Interventions Research Norwegian Institute of Public Health Oslo Norway

## Abstract

**Background:**

Systematic reviews are increasingly used to inform decision‐making in health, education, social care and environmental protection. However, decision makers still experience barriers to using reviews, including not knowing how findings might translate to their own contexts, and lack of collaboration with systematic review authors. The TRANSFER approach is a novel method that aims to support review authors to systematically and transparently collaborate with stakeholders to consider context and the transferability of review findings from the beginning of the review process. Such collaboration is intended to improve the usefulness and relevance of review findings for decision makers.

**Objectives:**

We aim to explore the user experience of the TRANSFER approach conversation guide, and in doing so gain a better understanding of the role and perceived value of stakeholder engagement in systematic reviews for informed decision‐making.

**Methods:**

We conducted four user tests of groups using the guide, organized around simulated meetings between review authors and stakeholders. Review authors led the meeting using the TRANSFER approach conversation guide. We audio‐recorded and observed the meetings, collected feedback forms and conducted semi‐structured interviews with review authors following the meeting. We analysed the data using framework analysis to examine the user experience of the TRANSFER approach conversation guide and of stakeholder engagement more generally.

**Results:**

Seventeen participants in four user groups participated in the user tests. Most participants were generally positive toward the structured approach using the conversation guide, and felt it would be useful in systematic review projects. We observed examples of misunderstanding of the terminology included in the guide, and received multiple suggestions for how to make the conversation guide more user friendly. We observed numerous challenges related to the hypothetical nature of a user test, including lack of familiarity with the review question/topic among participants and lack of preparation for the meeting.

**Conclusions:**

Review authors and stakeholders are positive toward using a structured approach to guide collaboration within the context of a systematic review. The TRANSFER conversation guide helps participants to discuss the review question and context in a structured way. Such structured collaboration could help to improve the usefulness and relevance of systematic reviews for decision making by improving the review question, inclusion criteria and consideration of transferability of review findings. The conversation guide needs to be modified to improve user experience. Further research is needed to explore stakeholder collaboration and the use of the TRANSFER conversation guide in systematic review processes.

## 1 BACKGROUND

Decision makers in the fields of health, education, social welfare, and environmental protection are increasingly aiming to draw on the best available research evidence to inform their decision‐making processes (Dicks et al., 2017; Gough et al., 2011; Lundahl et al., 2009; Yost et al., 2014). Systematic reviews, widely viewed as the best source of such evidence, are designed to make the available evidence easier to find and use (Gopalakrishnan & Ganeshkumar, 2013). However, barriers to use persist (Oliver et al., 2014; Tricco et al., 2016; Wallace et al., 2012). In this study, we present an evaluation of a novel approach to address two of the perceived barriers to the use of research evidence in decision‐making processes, specifically, collaboration between systematic review authors and decision makers and the consideration of context of review findings.

### 1.1 Barriers and facilitators to using systematic review findings in decision making

Methods that support the use of findings from systematic reviews in decision making have evolved significantly over the past decades. Databases where reviews are published have become more physically accessible (e.g., through sources like the Cochrane Library; www.cochranelibrary.com), publishers have developed more user‐friendly formats to present findings (e.g., Supporting Policy‐relevant Reviews and Trial (SUPPORT) summaries; Rosenbaum et al., 2011) and researchers have developed robust frameworks that help decision making groups, such as guideline panels, consider the most important decision criteria and related research evidence in their decision making processes (Evidence‐to‐Decision Framework (EtD) (Lavis et al., 2009; Moberg et al., 2018; Rosenbaum et al., 2018). However, a number of real and perceived barriers to the use of evidence in decision making remain, including people's lack of skills to find and interpret evidence, available time, and knowledge among decision makers regarding the function of systematic reviews (Innvaer et al., 2002; Oliver et al., 2014; Tricco et al., 2016). Studies have also identified activities that may facilitate the use of evidence, including improved consideration of context, and improved collaboration between stakeholders and researchers (Oliver et al., 2014; Rosenbaum et al., 2011; Tricco et al., 2016). For the purpose of this study, the term *stakeholders* refers to anyone with an interest in the findings from a systematic review, including patients and service users, patients and the public, health care practitioners, policymakers and other decision makers.

### 1.2 Considering context and the transferability of review findings

An intervention's implementation context may influence how it works (Shepperd et al., 2009). Context is therefore an important consideration when evaluating, or exploring experiences and perceptions, of an intervention in a systematic review. For the purpose of this study, *context* refers to ‘the multi‐level environment (not just the physical setting) in which an intervention is developed, implemented and assessed: the circumstances that interact, influence and even modify the implementation of an intervention and its effects’ (Munthe‐Kaas et al., 2020). Most systematic reviews include studies from different contexts that may vary according to geography, politics and/or culture (Lewin et al., 2017). Synthesized findings, based on data from studies that may have been conducted in a variety of contexts, may pose problems when people begin to consider the transferability (sometimes referred to as applicability, directness, relevance, etc.) of review findings to a local decision‐making context. Decisions makers may question whether findings from a systematic review are relevant for their context. There is general consensus in the literature that ‘the real or perceived failure of reviews to address relevant questions, contextualize the findings, or make actionable policy recommendations’ can inhibit the use of systematic reviews in evidence‐informed decision making (Chambers et al., 2011; Innvaer et al., 2002; Lavis et al., 2005; Petticrew, 2004; Sheldon, 2005). This is perhaps particularly relevant for interventions that are more complex and which are comprised of ‘a number of separate elements which seem essential to the proper functioning of the interventions although the “active ingredient” of the intervention that is effective is difficult to specify’ (BUK Medical Research Council). Improved collaboration with stakeholders, including decision makers, may be one way by which to address questions about context and transferability of review findings.

### 1.3 Collaboration between stakeholders and review authors in conducting reviews

Stakeholder engagement in systematic reviews has long been advocated by researchers and stakeholders alike (Cottrell et al., 2014; Lavis et al., 2005; Sakala et al., 2001; Wale et al., 2010). There is a perceived ‘gulf between decision makers and researchers, which prevent[s] the production of research from feeding into decision making processes’ (Orton et al., 2011). Contact and collaboration between researchers and decision makers has been touted as both a barrier when absent, and a facilitator when present, to the use of review findings in decision‐making. Numerous studies have extolled the potential benefits of stakeholder engagement, including production of more useful and relevant systematic reviews (Cottrell et al., 2014; Sakala et al., 2001; Wale et al., 2010) (Aloe et al., 2020; Pollock et al., 2018). Engaging with stakeholders is considered critical for defining and ensuring a common understanding of the review question and what it seeks to answer, understanding the review contexts and improving stakeholder buy‐in, as they may sit with a great deal of expertise which may otherwise be unavailable or difficult to obtain (Cottrell et al., 2014; Esmail et al., 2015). Despite this enthusiasm, there is little published literature evaluating stakeholder engagement in research activities generally (Esmail et al., 2015). There are however, numerous studies calling for training materials, and examples of how to support stakeholder engagement in systematic reviews (Pollock et al., 2018).

Specifically, there is currently a lack of practical guidance on how researchers can effectively collaborate with stakeholders to systematically and transparently consider context and the transferability of review findings (Burchett et al., 2018; Burford et al., 2013; Gruen et al., 2005).

### 1.4 The TRANSFER approach

TRANSFER approach aims to ‘support … collaboration between review authors and stakeholders from the beginning of the review process to systematically and transparently consider factors that may influence the transferability of systematic review findings’ (Munthe‐Kaas et al., 2020). The main tenets of the TRANSFER framework are: (a) close collaboration between review authors and stakeholders, starting with the development of the review question, and (b) recognition that various stakeholders' expertise and contributions in the development of the review question are necessary for improving our consideration of context in systematic reviews. The TRANSFER approach consists of guidance for review authors, including guidance on:

‐ how to organize a meeting with stakeholders to define the population, intervention, comparison and outcomes to be examined in the review (using the PICO template)
‐ how to discuss and prioritize factors that may impact how transferable the studies are to the specified context (transferability factors) (Munthe‐Kaas et al., 2020) (using the TRANSFER Conversation guide)
‐ how to define the local context of interest (using the TRANSFER Characteristics of context template), and;
‐ how to assess the transferability of a review finding from one context to another (i.e. will the intervention have the same effect/be experienced in the same way for people in the decision making context if the data comes from studies where the context is substantially different according to the identified transferability factor(s)?)

The TRANSFER approach is intended to help review authors to identify factors that may influence the transferability of a review finding to a local decision making context. Identified transferability factors should be considered to be hypotheses, and can include factors related to geopolitical climate or social or cultural values. Review authors can use the identified transferability factors to inform subgroup analyses later in the review to assess whether a review finding is likely to be transferable to the review context. See Munthe‐Kaas et al. (2020, 2019) for more details on the TRANSFER approach and how it was developed (Munthe‐Kaas et al., 2019, 2020). See Table 1 for an example illustrating how the TRANSFER approach could support review authors to systematically consider how contextual factors could influence the transferability of review findings to the context of interest.

**Table 1:** Example illustrating when context matters in a decision‐making process

**Example of when context matters**:
You are commissioned to conduct a systematic review on the effect of having a labour companion during birth in a medical facility. The review findings will inform global guidelines, and is intended to be adapted and used by individual countries. The review authors find that labour companionship has positive effects for mother and child. The review authors conduct separate subgroup analyses for high and middle‐income contexts and find no substantial differences in effect.
When the intervention is implemented across a variety of settings, it becomes evident that the positive effects from the review are not seen in all contexts. Decision makers in certain settings hypothesize that the review finding does not transfer to their settings because the context of their settings differs substantially from the contexts of the studies that contribute data to the review findings. Specifically, the studies included in the review come from contexts where women can choose their labour companions, where the birthing facility has space for labour companions, and where the tasks of birthing companions are related to emotional support for the mother. The decision makers point out that in their settings women do not have free choice over their labour companions, there is limited space in birthing facilities, and companions are expected to carry out different tasks. These three factors are not necessarily common to high or middle income countries and thus were not covered in the subgroup analysis.
By identifying these specific transferability factors early in the review process (rather than simply comparing high vs. middle‐income contexts), the review authors could have extracted data related to choice of labour companions, physical space in birthing facilities and tasks of labour companions from the included studies. They could have then conducted a subgroup analysis using the three factors discussed above which may have indicated different (degrees of) effects in different contexts. The decision makers would then have been better able to interpret and/or implement the intervention to their own context.
*This is a fictionalized example of a review finding from a qualitative evidence synthesis conducted by Bohren and colleague*s (2019).

### 1.5 User testing to explore user experience of the TRANSFER approach

User testing (also referred to as usability testing) refers to a research method where the goal is to expose areas of improvement in the design of a service, system or product to improve the products next iteration (Rosenbaum et al., 2008). Part of this method includes exploring users' experience of using a product, in this case the TRANSFER approach. The term ‘user experience’ refers to ‘all the aspects of how people use an interactive product’ (Alben, 1996). It is ‘a consequence of a user's internal state (predispositions, expectations, needs, motivation, mood, etc.), the characteristics of the designed system (e.g., complexity, purpose, usability, functionality, etc.) and the context (or the environment) within which the interaction occurs (e.g. organizational/social setting, meaningfulness of the activity, voluntariness of use, etc.)’ (Hassenzahl & Tractinsky, 2006). By exploring user experience of the TRANSFER approach, we hope to better understand user needs and contexts, make appropriate revisions, and attempt to optimize the methods described in the approach.

## 2 AIM

Our study aimed to explore the user experience of the TRANSFER approach conversation guide from the perspective of the systematic review team and the stakeholders who have an interest in using the findings from a systematic review. A secondary aim was to gain a better understanding of the role of stakeholder engagement in systematic reviews, and stakeholders perceived value of this engagement, for informed decisionmaking.

## 3 METHODS

### 3.1 User‐centred development

We employed a user‐centred design approach in this study. User‐centred design, sometimes called human‐centred design, is an approach to developing a product, service or system that puts the users' and other stakeholders' needs at the centre of the development (International Organization for Standardization, 2010). Two key characteristics of this approach are: (1) development that is based on an explicit understanding of users' needs and (2) an iterative process that entails creating or improving a prototype, exploring peoples' experiences of that prototype while carrying out tasks, identifying areas for improvements, and repeating the cycle.

### 3.2 User testing method

To understand users' needs related to TRANSFER, we explored their user experiences of the tool by combining several methods. At the core we drew on a method called ‘user testing’—a study design commonly applied in design development (Chisnell & Rubin, 2008) to understand people's ability to carry out desired tasks with a product or service.

We employed a ‘constructive interaction with think‐aloud’ method (Als et al., 2005; Kahler et al., 2000; Van den Haak et al., 2004). This involved participants working together using the TRANSFER approach while we observed and captured their concurrent think‐aloud reflections of what they were thinking and feeling. From now on, we will refer to these sessions as ‘group user tests’. We followed up these group user tests immediately with individual semi‐structured interviews employing retrospective think‐aloud and written participant feedback.

### 3.3 Participant selection

We used a purposive sampling method to identify participants. For each group user test we aimed to include at least: (a) two participants who had experience conducting systematic reviews (effectiveness reviews in three user tests, and qualitative evidence synthesis in one user test), and (b) two participants who were considered to be stakeholders in a review process (including policy makers, guideline panel members, other decision makers) and who were familiar with using the findings from or commissioning systematic reviews. We sent email invitations to systematic review authors from research institutes in Norway and policy makers from the Norwegian directorates of Health, Integration and Diversity, and Children, Youth and Family Affairs. Test participants were not rewarded financially but did receive reimbursement for travel costs when requested.

### 3.4 Data collection

Each group user test was made up of a meeting scenario, feedback forms and semi‐structured interviews with review authors.

#### 3.4.1 Before the user tests

Before each group user test, we sent the following documents and information via email to systematic review authors:

a. TRANSFER approach guidance for systematic review authors.
b. Document describing the background, review question, PICO (Population, intervention, comparison, outcomes), and inclusion criteria for a fictionalized adaptation of a systematic review.

We sent the latter document to the stakeholder participants. See supplementary material for the documents that were sent to participants.

#### 3.4.2 Collaborative meeting scenarios

We designed scenarios to resemble a typical meeting between review authors and stakeholders at the beginning of a systematic review process, with two systematic review authors and two decision makers from a Norwegian directorate. We instructed people to participate from the perspective of their assigned role (review author or stakeholder). For each meeting, we assigned one of the two following systematic review question to be discussed: ‘Welfare‐to‐work interventions and their effects on the mental and physical health of lone parents and their children’ (Gibson et al., 2017) and ‘Perceptions and experiences of labour companionship: a qualitative evidence synthesis’ (Bohren et al., 2019). We chose these two reviews as they examined interventions that we deemed relatively familiar for most people, and due to their social nature, could be influenced by context. We assigned the effectiveness review to groups with authors who had experience synthesizing quantitative data, and the qualitative review to authors with experience synthesizing qualitative data.

We held the meetings at the Norwegian Institute of Public Health. Our involvement was limited to providing an introduction, observing the meeting, and clarifying terms or concepts when necessary. We audio‐recorded and transcribed group user tests 2, 3 and 4.

The introduction included a description of the TRANSFER approach, the group user test method our research aims, and a question/answer session. Participants received and signed informed consent forms that gave us permission to use their feedback for improving TRANSFER and for research publication. Review authors led the simulated meeting according to steps in the guidance we provided beforehand: (1) briefly summarize the review question, and; (2) use the conversation guide to discuss possible factors that could influence the transferability of systematic review findings to the context specified in the review question, and the local (Norwegian) context. The first part of the TRANSFER approach, defining the PICO and review question, was not included in the group user test due to time restrictions.

#### 3.4.3 Think aloud approach

We employed a ‘concurrent think aloud’ approach whereby participants were encouraged to verbalize their thoughts at the same time working through the conversation guide together (Richardson et al., 2017). This involved each person reflecting their thoughts out loud as the group went through the various steps. During the semi‐structured interviews, we employed a retrospective think‐aloud technique, where participants drew on their memory of what had just transpired in the meeting.

#### 3.4.4 Observation

At least one researcher observed and made notes in each of the user tests.

#### 3.4.5 Feedback forms

Immediately following the meetings, participants filled out feedback forms. See Supporting Information: Appendix 2 for a copy of the feedback form used. The feedback forms were intended to reflect the facets of user experience from an adjusted version of Morville's Honeycomb model (see Table 2) (Rosenbaum, 2010).

**Table 2:** Facets of user experience according to an adjusted version of Morville's Honeycomb model (Rosenbaum, 2010)

Facet of user experience	Definition
Findability	Can users locate what they are looking for?
Accessibility	Are there physical barriers to actually gaining access, also for people with handicaps?
Usability	How easy and satisfying is this product to use?
Usefulness	Does this product have practical value for this user?
Credibility	Is it trustworthy?
Desirability	Is it something the user wants? Has a positive emotional response to?
Value	Does this product advance the mission of the organization behind it?
Affiliation^a^	Does the user feel that they are the appropriate audience for the product (i.e. is the product targeted towards me?)
Understandability^a^	Does the user understand the content of the product?

a Added by Rosenbaum 2010 (Rosenbaum, 2010).

#### 3.4.6 Semi‐structured interviews

We interviewed the review author participants immediately after they filled out feedback forms. We chose to only interview review authors because we wanted to understand how they experienced using the conversation guide to lead the meeting. We conducted semi‐structured interviews as they are a suitable method for exploring a set of known topics while allowing participants to raise new issues (Drever, 1995). At least one researcher observed the interviews. We audio‐recorded some of the sessions. The topics of the interview guide reflected the facets of user experience from an adjusted version of Morville's Honeycomb model (see Table 1) (Rosenbaum, 2010). See Supporting Information: Appendix 3 for the interview guide.

#### 3.4.7 Real‐life meeting observations

In addition to the group user tests, we also applied TRANSFER to a real‐life review process. Here, a research team (led by a colleague that had participated in a user test) that was in the process of planning a review invited one of the authors (H. M. K.) to help prepare a stakeholder meeting using the TRANSFER approach conversation guide to discuss context. The researcher provided pre‐meeting guidance and conducted non‐participatory observation of the meeting and took notes.

### 3.5 Analysis

We transcribed the audio‐recordings of interviews. A third party transcribed the audio‐recordings of two of the meetings and one researcher checked them for accuracy. We analysed data using the framework analysis method (Ritchie & Lewis, 2003). We used an a priori template of codes based on an adapted version of Morville's honeycomb model of user experience (minus the facets *accessibility* and *value*, which we deemed to be premature at this stage in the development of TRANSFER; see Table 3) as the basis for analyzing the user experience of the conversation guide (Boyatzis, 1998; Crabtree & Miller, 1999) (Rosenbaum, 2010; Morville, 2004; Fereday & Muir‐Cochrane, 2006). Through our analysis we identified many findings related to stakeholder engagement and other aspects of the meeting scenario that did not fit into the honeycomb model. We decided to use thematic analysis to organize these findings into themes (Boyatzis, 1998).

**Table 3:** Description of participants

User test	Role	Background
1	Review author	Male, Systematic review author, >5 years experience
1	Review author	Female, Systematic review author, >5 years experience
1	Stakeholder	Female, Senior advisor, Norwegian government department
1	Stakeholder	Female, Senior advisor, Norwegian government department
2	Review author	Female, Systematic review author, >5 years experience
2	Review author	Female, Systematic review author, >5 years experience
2	Stakeholder	Female, Senior advisor, Norwegian government department
2	Stakeholder	Female, Senior advisor, Norwegian government department
3	Review author	Female, Systematic review author, <5 years experience
3	Review author	Male, Systematic review author, <5 years experience
3	Stakeholder	Male, Senior advisor, Norwegian government department
3	Stakeholder	Male, Senior advisor, Norwegian government department
4	Review author	Female, Systematic review author, experience with qualitative evidence synthesis, >5 years experience
4	Review author	Female, Systematic review author, experience with qualitative evidence synthesis, >5 years experience
4	Stakeholder	Male, Systematic review author, experience with qualitative evidence synthesis, >5 years experience
4	Stakeholder	Systematic review author, experience with qualitative evidence synthesis, >5 years experience
4	Stakeholder	Associate professor, experience with qualitative evidence synthesis

Two researchers coded each of the documents. We discussed all of the codes and themes until consensus was reached. We did not need to bring in a third researcher to settle any disagreements. We actively looked for examples of contrasting and discrepant participant opinions/experiences. All of the data were coded and analysed in their original language. We translated quotations for use in this article.

### 3.6 Data privacy and ethical consideration

According to the Norwegian Centre for Research Data (nsd.no) online portal for determining registration of studies, this study did not warrant application for review by the data privacy committee https://nsd.no/personvernombud/en/notify/index.html (i.e. participants could not be identified in the data, and we did not save, analyse or use and sensitive or personal information). In Norway, this type of study falls outside of the remit of the Regional Committee for Medical and Health Research Ethics. In this case, it remains up to each institution to follow best ethical research practices. We followed ethical guidelines as outlined by The Norwegian National Committees for Research Ethics (Guidelines drawn up by The Norwegian National Research Ethics Committee for medical and health research).

## 4 FINDINGS

We identified a number of themes from the analysis of the group user tests which we have organized into the following broad categories: findings related to the user experience of the TRANSFER conversation guide, observations and experiences of stakeholder collaboration, participants' experiences unpacking the concepts ‘transferability’ and ‘context’, and results of the meeting scenarios. We also present findings related to our observation of the application of TRANSFER in a systematic review process. We will begin by presenting an overview of the participants.

### 4.1 Participant profiles

We included 17 participants in four group user tests conducted in Norway in 2018 and 2020. See Table 3 for an overview of relevant participant characteristics. For the remainder of this paper, *participants* refers to either stakeholders or review authors. In three of the group user tests the stakeholders were decision makers from Norwegian governmental departments that often commission systematic reviews and/or are involved in guideline processes health and social services. In one group user test, we were unable to engage real decision makers, so three review authors played the role of stakeholders. We will still refer to these participants as *stakeholders* throughout the findings section.

One of the review authors was familiar with the concept of the TRANSFER approach, and two review authors had been involved in the development of the approach, but did not have experience using the conversation guide. Three of the eight stakeholders had taken part in an earlier iteration of the TRANSFER approach but indicated to the research team that their experience with or knowledge of TRANSFER felt insignificant.

### 4.2 Findings related to users' experience of the TRANSFER conversation guide

In this section, we describe the users' experience of the TRANSFER conversation guide. We begin by describing general impressions and summarizing participants' feedbacks with a selection of quotes to illustrate their experiences. We then present a table that organizes participants' feedback into themes using a modified version of Morville's honecomb model (see Table 4) (Rosenbaum, 2010).

**Table 4:** Overview of themes related to the user experience of the TRANSFER conversation guide

Usefulness	Useful for structuring dialogue between review authors and stakeholders
	Helped to acknowledge and address assumptions and potential myths regarding context
	Helpful in refining the review question and inclusion criteria
	Potentially time consuming
	The format should be changed to emphasize that it is not a checklist
Desirability	Could be a useful tool in a review process
	Not convinced transferability of review findings is actually a problem for decision makers
Credibility	Improves interpretation of results
	Is in accordance with guidance on performing sub‐group analyses (defining subgroups a priori)
	Can improve review question and PICO
Usability	Perception of usability varies
	The format limited the discussion (easy to get stuck going line by line through the conversation guide)
	Structured otherwise disordered discussions
	Helped review authors end digressions
Understandability^a^	Too much researcher jargon, needs to be adapted for non‐researchers
Affiliation^a^	Language needs to be adapted for use in qualitative evidence syntheses
	Active participation by stakeholders and review authors
Findability	The PICO structure makes it easy to navigate the list
	Format should go from broad issues (environment, geopolitical) down to more specific issues (population)
	Concerns regarding whether review authors could find information regarding the identified transferability factors in included studies
Accessiblity	Not evaluated
Value	Not evaluated

a Added to the original model by Rosenbaum 2010 (Morville, 2004; Rosenbaum, 2010).

The participants appeared to have a generally positive response to the TRANSFER conversation guide:

[The TRANSFER conversation guide] would have been helpful in previous reviews (review author, user test 1 interview).

[The conversation guide] brought forth many relevant discussions and covered what was necessary (and maybe even more than that) (stakeholder, user test 3 feedback form).

Generally, we observed great variation in how easily participants appeared to be able to use the conversation guide document. One participant speculated that it would be easier to use with practice:

To a certain extent, one feels unprepared for this. It is good to be tuned in. It was good. It becomes a training exercise. If you have done this a few times with a specific problem, it will become easier (stakeholder, user test 2 meeting scenario).

However, participants expressed practical concerns regarding the usability of the conversation guide such as how much time it may take to use it, potential difficulty of assessing identified transferability factors, and whether all factors are equally relevant for each review.

Additionally, some participants found the format challenging. For example, several participants expressed concerns that the list style of the conversation guide could lead to overly detailed discussions. Participants suggested that the conversation guide should be simplified, that there should be more space for notes, and that it should be clearer that the list is meant as ‘prompts’ and not a checklist.

Several participants struggled with the terminology, or research jargon, used in the conversation guide. One participant commented that it would be helpful to have a translation of the conversation guide available in their own language.

Participants' suggestions for improving the guide included offering more examples and explanations for each potential factor, giving more detailed instructions, making the guide more flexible, adding a glossary of terms and including a more in‐depth discussion of the review question before embarking on the conversation guide.

In Table 4 we present an overview of the findings related to users' experience of the TRANSFER conversation guide organized in accordance with a modified version of Morville's honeycomb model (Rosenbaum, 2010).

### 4.3 How participants experienced stakeholder collaboration

In this section, we describe our observations of, and participants' own expressed experiences with, stakeholder collaboration during the simulated meeting. The findings described in this section are based on both the authors' observations of the user test, participants' voiced thoughts (using the think‐aloud technique) and feedback gathered from the semi‐structured interviews with review authors following the simulated meeting. There is therefore an over‐representation of feedback from review authors. We begin by presenting findings related to participants' experience of structured stakeholder collaboration and then present findings related to how the participants understood and perceived the goal of the TRANSFER approach in particular.

#### 4.3.1 Findings related to structured stakeholder collaboration

One of the central tenets of the TRANSFER approach is to offer practical guidance to review authors on how to collaborate with stakeholders. Based on our observations and written feedback, a structured approach to discussing context and transferability was beneficial and organized an otherwise potentially chaotic discussion:

I think this is really useful to have a main checklist where one can go through potentially important things (review author, user test 3 interview).

Furthermore, in some cases the participants recognized that the conversation guide helped them to uncover transferability issues that they may not otherwise have thought about:

And we may have, if we hadn't had this, I'm just thinking out loud now, if we hadn't had this conversation, and it'd just been up to you and me, we may have sort of invented … We may have put a lot less emphasis on, for instance, cultural expectations than more on whether the women were identical to average Norwegians, I don't know. Possibly (review author, user test 4 meeting scenario).

All participants actively participated in the meeting scenarios. Although the review authors usually led the discussions, there were instances where a stakeholder took initiative to move to the next factor in the conversation guide. One stakeholder expressed that they wished they had also received the conversation guide in advance, a document we thought would only be of interest to the review authors.

One participant noted that it could be valuable to include other types of stakeholders (such as practitioners) in meetings because their points of view could improve the usefulness of the conversation around context.

Although stakeholder collaboration is generally acknowledged in the literature as critical to the production of systematic reviews within informed decision making (Innvaer et al., 2002; Oliver et al., 2014; Tricco et al., 2016), one review author questioned that assumption:

[The conversation guide] can create a better dialogue with stakeholders, but is it a research question whether that is in fact useful in the end? (review author, interview after user test 2).

Participants also suggested that other aspects of the systematic review production might benefit from stakeholder collaboration at the beginning of the review process. A number of participants indicated that the conversation guide and meeting could help improve the way they considered the review question, the inclusion criteria, the subgroup analyses, interpret the results and possibly support GRADE/‐CERQual assessments:

Chaos during this meeting leads to a clearer PICO and review question … the PICO refinement is really valuable (review author, user test 1 meeting scenario).

We have a project with vague inclusion criteria but which is really dependent on context. For that topic area especially, it would have gone a lot smoother when it came to inclusion/exclusion of relevant studies (review author, user test 3 interview).

You could use [the identified transferability factors] to really sort of narrow down your inclusion/exclusion criteria or to guide your sampling (review author, user test 4 meeting scenario).

#### 4.3.2 How participants perceive and understand the purpose of the TRANSFER approach

However, in observing the user test meeting scenarios, we discovered that there was much apparent confusion regarding the goal of the TRANSFER approach, and the conversation guide specifically. It seemed difficult for both review authors and stakeholders to remember that the goal of the meeting was to identify transferability factors (i.e. factors that would make them concerned if data contributing to a review finding came from a different context then their decision‐making context):

I need a little reminder of what it is we are looking at and what exactly we are talking about because, um, we should, what we are looking for, is to find only what isn't comparable or transferable, is that it? (stakeholder, user test 3 meeting scenario).

I think if this is to be used by others, more detailed instructions will be needed, especially in terms of focusing on transferability rather than all modifying factors (review author, user test 4 feedback form).

Additionally, participants did not always understand what the identified transferability factors would be used for, for instance wonder if they would be used to inform inclusion criteria, or to predefine subgroup analyses. On the other hand, some participants not only grasped the purpose of identifying transferability factors, but also were able to suggest methods for assessing transferability of review findings using the transferability factors:

We are looking for factors that we think could influence the transferability of the effect? Many things can influence the effect, but we are looking at factors that could influence how the effect is in our context … we must think generally—can this factor have an effect on the transferability of the effect? And then think about special context or country. They must be able to influence effect in order to influence transferability (review author, user test 1).

Someone said it was difficult to know how health systems in other countries are. When we have an overview of included studies, and where they were conducted, can we divide them in two? Which systems are similar to the Norwegian system, and which are more similar to the American model? And then see if there are large differences between the two groups of studies? (review author, user test 2).

### 4.4 Participants' experiences unpacking context and transferability

One of the aims of the TRANSFER approach is to provide guidance to review authors on how to systematically and transparently consider contextual factors that may influence the transferability of review findings. Our data showed how TRANSFER could support this process, while uncovering some of the areas where people struggled.

#### 4.4.1 Getting past assumptions to more fine‐tuned understandings

Context may mean different things to different people. TRANSFER provides an explicit definition: ‘the multi‐level environment (not just the physical setting) in which an intervention is developed, implemented and assessed: the circumstances that interact, influence and even modify the implementation of an intervention and its effects’ (Munthe‐Kaas et al., 2020). Attention is directed to the specific factors related to the implementation environment, and not just the country or setting as a whole (e.g. Norway is not a contextual factor, but the multi‐party political system present in Norway could be a contextual factor).

We observed that the conversation guide often helped participants to steer away from generalizations such as ‘Scandinavia is different than the United States of America’ and focus on how two contexts may differ specifically.

We should be focusing on the transferability of a study context to our context—not from one country to another (review author, user test 1).

Although participants often used examples of how another country may be different from their own context, they did this with respect to a specific transferability factor. In this example the participants were discussing if health or social welfare systems could influence transferability:

And here the outcome will be that you have to pay out of pocket to go to the doctor, or be covered by insurance. Like in the USA for example it can be really expensive to go to the doctor versus in Norway where it is almost free (review author, user test 3 meeting scenario).

Participants also pointed out that context may not always be important:

The difference between an intervention lasting three or six weeks in Italy is the same as it lasting three or six weeks in Norway, it is the same with frequency (review author, user test 1 meeting scenario).

Several participants appeared to acknowledge that contexts could be conceived of as much narrower than the country level:

You have a high‐income setting, but a study can come from a much more narrow geographical area like the Bronx that has certain characteristics, or other times it is wider, for example the whole of New York City. Then you will get a lot of different populations … (review author, user test 3 meeting scenario).

We observed that participants began thinking about how to define their own context according to the transferability factors being discussed:

It is maybe the tradition in our context that people move back home or closer to one of the parents' family? (discussing social support, review author, user test 3 meeting scenario).

#### 4.4.2 Common problems with the concept of context

However, there were also examples of confusion regarding the concept of *context*. For example, we often observed that the conversation would digress into discussions of how the effect of the intervention may be different for various subgroups of participants, which is not necessarily related to context. One participant explicitly expressed their confusion when trying to define the decision‐making context (which could include factors related to demographics of the population, or characteristics of the intervention):

I'm a little confused by the use of context. Context I think refers to the environment (review author, user test meeting scenario 2).

We also observed that participants often made assumptions regarding context that may not necessarily be true. In one user test the participants referred back to the inclusion criteria ‘high income countries’ numerous times to say that the studies would likely be similar to Norway because they came from high income countries, but at one of the participants pointed out that there can be differences within the group ‘high‐income countries’ (stakeholder, user test meeting scenario 4).

Furthermore, several participants noted that many primary studies do not include the kind of information related to many of the potential transferability factors in the conversation guide:

The studies don't always describe the context of the policies and rules underlying the intervention, because it is sort of a given in the country where the study is done. And it may be a lot of work … (review author, user test 4 meeting scenario).

#### 4.4.3 Transferability and qualitative research

Participants briefly discussed the appropriateness of considering transferability (generalizability) of qualitative research, however one participant proposed that this debate was immaterial in the current circumstances:

I know exactly which discussion you mean … but I think, the fact that we're even doing a review is kind of almost past that, I mean, just the idea of doing a systematic review has already sort of, acknowledged that there is some value in, in, in generalising or in synthesising … in a way, I would've thought the transfer addresses this issue, because actually thinks about it in a more structural, intelligent way … (review author, user test 3 interview).

There were, however, concerns that the conversation guide terminology and subcategories were not completely tailored for application to qualitative research.

This is a very effectiveness review‐list, and we're answering more qualitative … so words like ‘compliant’ and ‘intervention’ (stakeholder, user test 4 meeting scenario).

### 4.5 Summarizing the meeting scenarios

In this section we describe two issues related to the results of the meeting scenarios. First, we discuss challenges we observed related to the hypothetical nature of the simulated meetings. Next we present and compare the results of the individual meeting scenarios from each user test.

#### 4.5.1 Challenges related to the hypothetical nature of meeting scenarios

We observed a number of challenges related to the conduct of the actual user test, rather than the TRANSFER approach. Most participants found it difficult to discuss a fictitious review question on a unfamiliar topic.

It is difficult to say theoretically … This [checklist] is a bit difficult I think, when it is so abstract. But when we get to see the review, maybe we can think then (review author, user test 2 meeting scenario).

and again, I think it is difficult to prioritize before you know what is most relevant. I don't know if I would have done this exercise without any preparation. Just would have had a short chat, with a kind of guide I would have been able to see what the challenges would be before I spent more time (review author, user test 2 meeting scenario).

Only one of the eight systematic review participants appeared to have read the guidance or conversation guide beforehand. This lack of preparation was evident both in how review authors led the meeting, used the conversation guide, asked questions and gave feedback.

We would have read more about this and thought through the PICO and the transferability factors. Discussed internally what we would need to remember to ask about (review author, user test 2 meeting scenario).

A stakeholder participant hoped that review authors would be better prepared in a real‐life process:

[I think … that if this approach is going to be used. Will you [referring to review authors] be trained on it? There are a lot of factors here. Then you would be more tuned in] (stakeholder, user test 2 meeting scenario).

#### 4.5.2 Presentation and comparison of the results of the meeting scenarios in each user test

We present an overview of the individual meeting scenarios in Table 5. Generally, we observed much variation in how the meeting scenarios were conducted. Despite discussing the same review question (in three of four scenarios), and having comparable professional backgrounds (i.e. review authors or decision makers), the results of the meeting scenarios differed with respect to:

‐ Length of time spent on reviewing the PICO and review question
‐ Length of time spent on each factor
‐ Which factors were discussed
‐ Level of detail of discussion
‐ Prioritized transferability factors

**Table 5:** Overview and output of meeting scenarios using two pre‐defined review questions

User test	Participants	Review question	Conversation guide (see Supporting Information: Appendix 1)	Prioritized transferability factors
1	2 systematic review authors	What are the effects of Welfare‐to‐Work interventions on mental and physical health and employment and income for lone parents and their children living in high‐income countries?	Version 1	1. Social acceptability (including religion)—stigma related to use of welfare services
	2 stakeholders			2. Who is implementing the intervention?
				3. Usual care was discussed multiple times
2	2 systematic review authors	What are the effects of Welfare‐to‐Work interventions on mental and physical health and employment and income for lone parents and their children living in high‐income countries?	Version 1	1. Participant characteristics
	2 stakeholders			2. Welfare system and quality of usual services
3	2 systematic review authors	What are the effects of Welfare‐to‐Work interventions on mental and physical health and employment and income for lone parents and their children living in high‐income countries?	Version 2	1. Temporal context
	2 stakeholders			2. Health and welfare system
				3. Participant characteristics (e.g., education, single fathers vs. mothers)
				4. Presence of co‐existing/alternative interventions
				5. Outcomes—defined and measured differently, length and intensity of follow‐up
4	5 systematic review authors (2 playing role of stakeholders)	What are the perceptions and experiences of women, partners, community members, healthcare providers and administrators, and other key stakeholders who have experience with a labour companion?	Version 1	1. Is the institutional support for having access to companions similar to that in Norway (are policies and culture in place)?
				2. Are the tasks expected of the companions similar to those that the Norwegian companions would carry out?
				3. Are the physical conditions similar for companions?
				4. Are the companions similar to companions found in Norway?

However, there were a number of commonalities across meeting scenarios, including:

‐ Each group finished going through all of the factors in the conversation guide
‐ Participants were poorly prepared (to be discussed below)
‐ All participants contributed actively to the conversation

According to the feedback forms, most participants found the meeting scenario to be ‘very well suited to my team and me’.

Finally, although the participants in the meeting scenarios came up with different prioritized factors, many of the discussions had similarities. For example, there was a lot of discussion around usual care (or the quality and comprehensiveness of services in different contexts) and how this could influence the transferability of the findings. Each group of participants also discussed participant characteristics (to varying degrees of specificity).

### 4.6 Observation of application of TRANSFER in a systematic review process

As previously described, we also applied TRANSFER to a real‐life setting. When we compare the findings from the user tests with the real‐life example, we notice a number of key differences:

‐ The lead author recruited participants who had personal and/or professional experience with the phenomena of interest (the intervention) as opposed to participants who were unfamiliar with the topic
‐ The lead review author used the conversation guide as starting point to develop questions specific to the review topic and the meeting participants as opposed to going through the TRANSFER conversation guide line by line during the meeting. Before the meeting, the review author chose eight of what they considered the most relevant and important transferability factors to discuss. We re‐worded these transferability factors into questions that could be more easily understood by stakeholders. Examples of the question she used (adapted so as to protect the privacy of the participants) are:
  ∘ Do you think there is any difference between the way population X and population y communicate with each about this topic now compared to 10 or 20 years ago?
  ∘ Do you think there is any difference between the way population x and population y communicate with each about this topic in other parts of the world? For instance, in countries with very different income levels from your own?

The above questions were based on specific transferability factors but were worded in a way as to encourage discussion that could perhaps include topics related to other transferability factors as well. The review authors only participated in the discussion to ask clarifying questions or move the discussion forward. They did not participate in the actual discussion of transferability factors. Furthermore, all participants actively engaged in the discussion and we even observed interactions between participants that were not facilitated by the review author (e.g., one participant asking another participant how the issue was in their context).

## 5 DISCUSSION

This is one of the few studies we are aware of that attempts to explore the perceived value of a specific approach for engaging with stakeholder and evaluates guidance for review authors on how to engage with stakeholders (Esmail et al., 2015). The findings from this study emphasize the potential value of discussing the review question before beginning a discussion on transferability and context. Such a discussion could help to ensure a common understanding of the review question, PICO, inclusion criteria and the decision makers' context among review authors and stakeholders before the review process commences. Although the meetings scenarios resulted in different lists of prioritized factors, there were some similarities across the groups. Furthermore, participants in this study were generally positive towards using a structured approach to discussing transferability and context in collaboration with stakeholders.

### 5.1 Considering context and transferability

When provided with a list of transferability factors to consider, review authors and stakeholders are able to discuss context in a more thoughtful and nuanced manner, moving beyond country level to actually defining what it is that may make studies from a certain context less transferable to another context. Such a considered approach to context would hopefully facilitate the use of review findings by decision makers by producing reviews that are more useful and relevant for a decision makers' context (Innvaer et al., 2002; Oliver et al., 2014; Tricco et al., 2016). Additionally, such a systematic and transparent consideration of context may be able to inform assessments of the GRADE/‐CERQual domains of *indirectness* and *relevance*. Some papers have suggested using a Delphi approach as a means to collaborate with stakeholders and collect information on transferability from stakeholders (Nasser et al., 2012; Wang et al., 2006).

The user tests also revealed a fundamental issue related to the TRANSFER approach related to transferability factors. Are transferability factors merely a subset of effect modifiers? In this case, should we consider expanding the remit of TRANSFER from only considering transferability factors, to identifying all potentially important effect modifiers related to the review question? This concept needs further investigation and thought.

### 5.2 Stakeholder collaboration

Stakeholder collaboration in itself is widely regarded as an important way to ensure the use of systematic review findings in informed decision making (Innvaer et al., 2002; Oliver et al., 2014; Tricco et al., 2016). Findings from this study indicate that collaboration between review authors and stakeholders could lead to a more clearly defined review question and to a more structured discussion of potential transferability factors. Furthermore, a *structured* approach to stakeholder collaboration may encourage more active stakeholder participation by creating a space where it becomes evident to the stakeholder that their expertise is valuable, appreciated and has the potential to improve the relevance of the systematic review findings to their decision‐making context. Such stakeholder participation may not only affect the *actual* relevance of the review findings, but may also potentially influence the stakeholder's *perception* of the relevance of the review findings.

### 5.3 Improving user experience of the TRANSFER conversation guide

Although participants were generally positive towards the use of the TRANSFER approach, the group user tests uncovered several format and content issues that need to be addressed to improve the TRANSFER conversation guide. In particular, we need to find ways to convey the usefulness of the TRANSFER approach for people who are unfamiliar with it. Although people indicated that use would become easier with experience, it is important to resolve challenges facing first time users, such their experience of length and density of the conversation guide, need for more examples/explanations, and facilitate easy meeting preparation. Furthermore, given the complex nature of discussions on context and transferability, translations of the conversation guide (and guidance for review authors) to languages other than English would likely be helpful. Finally, since only one review author read the guidance for review authors that we sent before the user test, we must explore whether the length or density of the guidance for review authors is a hindrance to its use, or whether it was difficult for participants to find time to prepare for a user test.

### 5.4 TRANSFER and qualitative evidence syntheses

The findings from this study indicate that review authors were positive toward the use of TRANSFER in qualitative evidence syntheses. We acknowledge that there is a wider debate on the appropriateness of assessing transferability (generalizability) of qualitative research (Finfgeld‐Connett, 2010). However, given the underlying beliefs of the authors (and participants in this study) that qualitative research should be synthesized to inform decision making, the debate regarding generalizability is immaterial. Within the ‘subtle realist’ position (social reality is not completely constructed), synthesis can provide a greater understanding of a phenomenon than a single primary study (Hammersley, 1992). However, as with reviews of effectiveness, it is important to consider and assess factors that could influence the transferability of review findings to a decision‐making context.

Moreover, the participants proposed that identified transferability factors could also be used to inform inclusion criteria and the sampling strategy in a synthesis. There appear, however, to be three important issues to explore before incorporating TRANSFER into reviews of qualitative evidence: First, how does the TRANSFER approach fits within the GRADE‐CERQual Approach (Lewin et al., 2015). Second, how could TRANSFER fit with qualitative syntheses that use more interpretive synthesis methods. Third, how can the language in the conversation guide be adapted for use with qualitative research.

### 5.5 Challenges related to group user testing

The advantage of involving multiple participants in each group user test was that it more closely resembled a meeting between review authors and stakeholders. The ‘think aloud’ protocol was also considered particularly useful as it allowed us more insight into the individual thoughts of each participant within the group, and not just the overall interaction between participants.

A drawback of involving multiple participants in a group user test, however, is that it was difficult and time consuming to transcribe the data, and analyse the findings. Furthermore, it was very difficult to compare and contrast between group user tests with different participants because the discussions were very connected to the dynamics of the specific group of participants.

We also encountered challenges related to the nature of group user tests more generally. It was difficult to recruit participants; therefore in one session review authors took on the role of stakeholders, rendering that data less representational and therefore weaker. During the group user tests, participants were often frustrated or confused due to the unfamiliarity of the review question being discussed, and participants did not prepare in the way that (we hope) they would for a real meeting with stakeholders.

### 5.6 Observing application of TRANSFER in a systematic review process

Few of the challenges related to the TRANSFER conversation guide that we uncovered during the group user tests were present when we observed the application of TRANSFER In a systematic review process. This indicates that many of the issues that arose during group user tests may be addressed by better preparation of the review authors adapting the conversation guide to the topic and participants, and discussing a topic that is relevant for the participants.

### 5.7 Future research

The findings from these user tests resulted in substantial changes to the format and language used in the TRANSFER Conversation guide (see above). We also realized the importance of including a discussion of the review question at the beginning of the meeting. We would like to test the refined version of the conversation guide, well as the PICO template with a wider range of review authors and stakeholders (e.g., different languages, settings, topical background, and experience levels). We would also like to continue observing the use of the TRANSFER approach in systematic review processes.

Some of the findings from this study can be used as evaluation measures in future studies to evaluate the experience and/or effect of stakeholder participation. For example, future studies could explore:

- The perceived benefits and challenges of a structured approach to stakeholder collaboration
- How the results of the TRANSFER approach are used in decision‐making after reviews are completed (e.g., compare how decision makers perceive the usefulness and/or relevance of reviews findings from reviews with and without the TRANSFER approach)
- Similarities and differences in which transferability factors are prioritized for a specific review question between groups of review authors and stakeholders from different contexts
- Other uses for the TRANSFER approach, such as informing inclusion criteria, supporting GRADE assessments of indirectness or GRADE‐CERQual assessments of *relevance*.

### 5.8 Researcher's role

The data for the study was collected and analysed by two researchers (the two first authors) who developed the TRANSFER approach. The researchers are experienced systematic review authors, familiar with conducting both reviews of effectiveness and qualitative research. At the time of the user tests, the researchers were colleagues of, or had previously engaged with most of the participants (review authors and stakeholders) at some point before the user test.

Our involvement in developing TRANSFER and familiarity with some of the participants may have influenced both the feedback given by participants, and how we analysed the data. On the other hand, our intimate knowledge of the TRANSFER approach put us in a unique position to understand: (a) where the conversation guide fell short of its intended aims, and (b) the nature of the participants' misunderstandings or problems. Likewise, participants' familiarity with us may have reduced the stress they felt while using a tool for the first time under observation.

### 5.9 Strengths and limitations

The strengths of this study are the use of multiple methods of data collection to triangulate data, the meeting scenario was organized to resemble a real meeting between review authors and stakeholders, and the use of the honeycomb model of user experience to interpret the findings. Furthermore, by including findings from our observation of a real‐life application of the TRANSFER approach in this study, we are able to explore which of the challenges identified in our analysis may be related to user testing rather than the TRANSFER approach. However, this study has the following limitations:

‐ A small number of user tests.
‐ Homogeneity among participants: Participants all came from the same geographical area, with review authors from the same institution.
‐ Simulated meeting scenarios, resulting in use of topics that participants were not necessarily experts in, and people not preparing for the meeting as much as they otherwise likely would have.
‐ The first part of the TRANSFER approach, defining the PICO and review question, was not included in the group user test due to time restrictions.

## 6 CONCLUSION

### 6.1 Implications for practice

The findings from this study indicate that review authors and stakeholders may find a structured approach to collaboration beneficial when considering context and transferability of review findings. The results of such structured discussions with stakeholders may be included in a systematic review to:

- provide rationale for inclusion criteria and subgroup analyses;
- discuss issues related to transferability; and,
- support GRADE/‐CERQual assessments of *indirectness* and *relevance*.

The goal of the TRANSFER approach, as well as the concepts *context* and *transferability* need to be clarified for review authors and stakeholders intending to use the TRANSFER approach. The TRANSFER conversation guide needs to be modified with respect to format and language to improve user experience. Furthermore, meetings with stakeholders are likely to be more productive when review authors are familiar with the conversation guide, and all participants are adequately prepared for the meeting.

Using a conversation guide when collaborating with stakeholders may lead better collaboration between review authors and clients, better review questions, clearer inclusion criteria and improved consideration of context. The TRANSFER approach conversation guide appears to be equally relevant for review teams undertaking qualitative evidence synthesis and reviews of effectiveness.

## AUTHORS' CONTRIBUTIONS

H. M. K. conceived of and designed the study, conducted the user testing, data analysis and interpretation of the results and drafted the manuscript. H. N. helped design the study, recruited participants, conducted the user testing and gave feedback on the data analysis, interpretation and the manuscript. User tests 2, 3 and 4 were transcribed using a professional service. S. R. advised on the study design and conduct. All authors read and approved the final manuscript.

## ETHICS DECLARATION

No ethics clearance was obtained for this study, as it is outside the remit of the Norwegian Health Research Law. Participants in the user tests were informed of the purpose of the study, and provided written consent to participate. According to the Norwegian Centre for Research Data (nsd.no) online portal for determining registration of studies, this study did not warrant application for review by the committee https://nsd.no/personvernombud/en/notify/index.html.

## COMPETING INTERESTS

H. M. K. and H. N. developed the TRANSFER approach. H. M. K. is a coauthor of the GRADE‐CERQual approach and lead co‐ordinator of the GRADE‐CERQual coordinating group. HN and SR have no competing interests.

## Supporting information

Supporting information.
