# Supplementary material for: User experiences of structured stakeholder engagement to consider transferability: The TRANSFER approach
Source: Campbell Syst Rev. 2022 Oct 17;18(4):e1284. doi: 10.1002/cl2.1284 (PMC9577259; doi:10.1002/cl2.1284)
Supplement: Supplementary file 1 — Supporting information. [file CL2-18-e1284-s001.docx]

## Appendices

### Appendix 1. TRANSFER approach conversation guide

#### Appendix 1.1. Conversation guide v1

| Category | Subcategory | Transferability factors to consider | Notes |
| --- | --- | --- | --- |
| Population | Participants | Participant characteristics (demographic characteristics, level of education, etc.) |  |
|  |  | Participant compliance levels |  |
|  |  | Previous exposure to current or other similar programmes |  |
|  |  | Availability of personal support for participants |  |
|  |  | Characteristics of illness / condition and comorbidities at participant level |  |
|  |  | Participant acceptability and preferences |  |
|  |  | Participant need for / access to information |  |
| Intervention | Intervention characteristics | Intervention components (details of intervention stages, phases, elements) |  |
|  |  | Category / status of intervention (policy, practice, program, guideline) |  |
|  | Intervention delivery | Duration of the intervention |  |
|  |  | Frequency / intensity of the intervention |  |
|  |  | Mode of delivery (physical setting, etc.) |  |
|  |  | Materials / manual - availability, characteristics, etc. |  |
|  |  | Standard procedures for the intervention in a real life setting |  |
|  |  | Possibility for tailoring intervention |  |
|  |  | Source of payment for receipt of intervention |  |
|  |  | Implementation fidelity |  |
| Implementation | Service providers | Number of service providers |  |
|  |  | Service provider characteristics |  |
|  |  | Type/position/status of service provider |  |
|  |  | Skills of service providers |  |
|  |  | Training of service providers |  |
|  |  | Monitoring, supervision and support of service providers |  |
|  |  | Service provider compliance |  |
|  |  | Factors that affect motivation of service providers |  |
|  | Implementing organization | Who is implementing organization? |  |
|  |  | Size and structure of the implementing organization |  |
|  |  | Implementing organization culture |  |
|  |  | Organizational policies |  |
|  |  | Implementing organization service systems |  |
|  |  | Implementing organization - financing |  |
|  |  | Implementing organization – interagency working relationships |  |
|  |  | Motivation of implementing organization |  |
|  |  | Essential resources needed/available |  |
|  |  | Communication and/or endorsement regarding intervention and implementation |  |
|  |  | Ease of implementation of intervention (feasibility) |  |
|  |  | Evolution / sustainability of intervention |  |
| Comparison intervention |  | Quality of comparison intervention |  |
|  |  | Characteristics of usual services |  |
|  |  | Standard procedures for comparison condition |  |
| Outcomes |  | Characteristics of outcome measurement |  |
|  |  | Costs |  |
|  |  | Length and intensity of follow-up |  |
|  |  | What are client important outcomes? |  |
| Environment | Temporal context | Temporal context |  |
|  | Political context | Political acceptability |  |
|  | Systems context | Health systems arrangements |  |
|  | Social context | Local professional/Expert opinion |  |
|  |  | Acceptability – community (e.g. religion) |  |
|  |  | Community (perceived) need |  |
|  |  | Social context – general (e.g. post-conflict, famine, etc.) |  |
|  | Other interventions | Alternative interventions |  |
|  |  | Co-interventions |  |
|  | Conditions for implementation | Conditions for implementation (geography, climate, etc.) |  |

#### Conversation guide v2

| **TRANSFER Factor** | **Would you be concerned if data come from contexts where…** | **Example** | **Notes** |
| --- | --- | --- | --- |
| **Environmental context** | | | |
| Temporal context | … the data was collected at a different point in time? | e.g., studies conducted before 2000 |  |
| Geo-political context | … the geographical, political or economic context is different? | e.g., studies conducted in post-conflict settings, settings where there is famine, high income settings, democratic settings, settings with colder/warmer temperatures, rural or urban settings |  |
| Health or welfare system context | …the health or welfare system is arranged differently? | e.g., free versus fee-based primary health care, comprehensive vs. limited family welfare services |  |
| Local professional/Expert opinion | … local professional/expert opinions are different? | e.g., experts are explicitly in favour or/against the intervention |  |
| Community acceptability | … the local community has a different level/degree of acceptability for the intervention or the condition being addressed by the intervention? | e.g. religious reasons, ethical reasons, other social reasons |  |
| Existence of alternative and/or co-existing interventions | … participants are exposed to alternative or supplemental interventions while participating in the intervention under examination? | e.g. contexts where all parents of small children are provided with free family counselling at the same time as they participate in a study where the intervention is online counselling for families with small children |  |
| **Participants** | | | |
| Participant characteristics | …participants are different with respect to demographic characteristics, level of education, etc.? | e.g., studies on participants older/younger than those in your context, contexts with a different gender ratio, |  |
| Participant compliance | …participants are different with respect to how well they follow instructions? | e.g., studies on pedestrian interventions to improve traffic safety in contexts where people are more/less likely to follow traffic rules |  |
| Availability of personal support for participants | …participants have different access to personal support networks? | e.g., contexts where families live close by vs. individualistic cultures |  |
| Characteristics of illness / condition and comorbidities | …participants’ condition or illness and comorbidities are different? | e.g., studies of an illness show that people in some contexts experience symptoms differently than people in other contexts |  |
| Participant acceptability and preferences | …participants level of acceptability and/or preferences regarding interventions/treatment, etc. are different? | e.g., studies of interventions from contexts where participants prefer to be called into versus make their own annual appointments |  |
| Participant need for / access to information | …participants have a different need for/access to/expectation of information? | e.g., studies from contexts where participants have a greater expectation of receiving comprehensive and detailed information regarding their treatment/intervention |  |
| **Intervention** | | | |
| Details related to the intervention | … the intervention components/stages/phases/elements are routinely/consistently differ from your context? | These issues may be covered while defining the review question and covered under inclusion/exclusion criteria in some cases. |  |
|  | …the intervention has a different duration, frequency, intensity? | These issues may be covered while defining the review question and covered under inclusion/exclusion criteria in some cases. |  |
|  | …the intervention is delivered in a different setting? | These issues may be covered while defining the review question and covered under inclusion/exclusion criteria in some cases. |  |
|  | …the availability and/or characteristics of materials/manuals for delivering the intervention is different? | These issues may be covered while defining the review question and covered under inclusion/exclusion criteria in some cases. |  |
|  | …the intervention is delivered differently than it would be in a “real life setting”? | e.g., laboratory/efficacy studies |  |
|  | …the intervention has been tailored? | These issues may be covered while defining the review question and covered under inclusion/exclusion criteria in some cases. |  |
|  | …the intervention is not delivered according to how it should be (i.e. implantation fidelity)? | e.g., the study authors describe clear deviations from how the intervention is intended to be developed (other checklists could be helpful here) |  |
| Category / status of the intervention | … the intervention is categorized differently? | e.g., policy, practice, programme, guideline |  |
| Implementation of the intervention | …the intervention is delivered by service providers who differ from those in your setting? | e.g., number of service providers, characteristics of service providers, such as training or skill level or type/status of service providers’ position, their compliance with implementation directions, any other factors that may influence their motivation to implement the intervention, such as religious beliefs, cultural background or support from leadership/colleagues? |  |
|  | …the intervention is implemented by an organization that differs from those that would be expected to implement the intervention in your setting? | e.g., type of organization, size/structure, culture, policies, service and financing systems, interagency working relationships, available/allocated resources, communication/endorsement of intervention, evolution/sustainability of intervention |  |
| **Comparison intervention** | | | |
|  | …the quality or comprehensiveness of the comparison intervention is different? | This is likely to be important for the transferability of most interventions |  |
|  | …”usual services” is different with respect to quality, comprehensiveness or content? | This is likely to be important for the transferability of most interventions |  |
| **Outcomes** | | | |
|  | …the way an outcome is defined or measured is different, including length and intensity of follow-up? | e.g., culturally different scales to measure quality of life, long-term versus short-term follow-up |  |
|  | …the way an outcome is prioritized (by clients/patients) is different? | e.g., patient-important outcomes |  |

### Appendix 2. Feedback form

**Feedback form for TRANSFER approach guidance and templates**

Thank you for taking the time to provide feedback. It will help us to improve guidance and templates for future users.

If you have any further questions please contact us at [heather.munthe-kaas@fhi.no](mailto:heather.munthe-kaas@fhi.no).

| **Date:** |  |
| --- | --- |
| **Role/position (stilling):** |  |
| **Do you have previous experience with/knowledge of the TRANSFER approach (specify)?** |  |


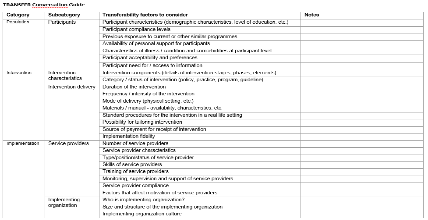
**TRANSFER Conversation Guide**

This form is first and foremost for collecting answers to the “why/why not” (identifying helpful features and problems) and “what could be improved” questions (collecting ideas about solutions). The check boxes are to make sure we understand the gist of your comments, and to frame suggestions in light of your overall experience.

| **I found the conversation guide easy to use** | Totally  disagree | 🞎 🞎 🞎 🞎 🞎 | Totally  agree |
| --- | --- | --- | --- |
| *Why/why not:* |  | | |
| *What could be improved:* |  | | |
| **I found the conversation guide content useful.** | Totally  disagree | 🞎 🞎 🞎 🞎 🞎 | Totally  agree |
| *Why/why not:* |  | | |
| *What could be improved:* |  | | |
| **I found the conversation guide format useful.** | Totally  disagree | 🞎 🞎 🞎 🞎 🞎 | Totally  agree |
| *Why/why not:* |  | | |
| *What could be improved:* |  | | |
| **I found the conversation guide language/instructions clear and understandable.** | Totally  disagree | 🞎 🞎 🞎 🞎 🞎 | Totally  agree |
| *Why/why not:* |  | | |
| *What could be improved:* |  | | |
| **The conversation guide was very well suited to my team and me.** | Totally  disagree | 🞎 🞎 🞎 🞎 🞎 | Totally  agree |
| *Why/why not:* |  | | |
| *What could be improved:* |  | | |


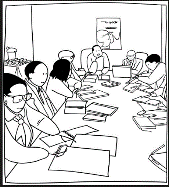
**Overall – meeting between stakeholders and review authors**

This form is first and foremost for collecting answers to the “why/why not” (identifying helpful features and problems) and “what could be improved” questions (collecting ideas about solutions). The check boxes are to make sure we understand the gist of your comments, and to frame suggestions in light of your overall experience. http://www.savyra.com

| **I found the meeting easy to participate in** | Totally  disagree | 🞎 🞎 🞎 🞎 🞎 | Totally  agree |
| --- | --- | --- | --- |
| *Why/why not:* |  | | |
| *What could be improved:* |  | | |
| **I found the meeting content useful.** | Totally  disagree | 🞎 🞎 🞎 🞎 🞎 | Totally  agree |
| *Why/why not:* |  | | |
| *What could be improved:* |  | | |
| **I found the meeting format useful.** | Totally  disagree | 🞎 🞎 🞎 🞎 🞎 | Totally  agree |
| *Why/why not:* |  | | |
| *What could be improved:* |  | | |
| **I found the meeting language/instructions clear and understandable.** | Totally  disagree | 🞎 🞎 🞎 🞎 🞎 | Totally  agree |
| *Why/why not:* |  | | |
| *What could be improved:* |  | | |
| **The meeting was very well suited to my team and me.** | Totally  disagree | 🞎 🞎 🞎 🞎 🞎 | Totally  agree |
| *Why/why not:* |  | | |
| *What could be improved:* |  | | |

### Appendix 3. Semi-structured interview guide

1. How easy was the conversation guide to use?
2. How useful was the conversation guide?
3. How useful is the TRANSFER approach?
4. How useful was the meeting with stakeholders?
